# Prognostic role of the pretreatment C-reactive protein/albumin ratio in gastric cancer: A systematic review and meta-analysis

**DOI:** 10.1097/MD.0000000000019362

**Published:** 2020-03-06

**Authors:** Xuanxuan Yang, Xing Song, Luo Zhang, Changping Wu

**Affiliations:** a Department of Tumor Biological Treatment; b Jiangsu Engineering Research Center for Tumor Immunotherapy; c Institute of Cell Therapy, The Third Affiliated Hospital of Soochow University, Jiangsu Changzhou 213003, China.

**Keywords:** C-reactive protein/albumin ratio, gastric cancer, meta-analysis, prognosis

## Abstract

**Background:**

In recent years, several studies have investigated the prognostic role of the pretreatment C-reactive protein/albumin ratio (CAR) in gastric cancer and yielded conflicting results. Therefore, we performed a meta-analysis to assess the prognostic role of the pretreatment CAR in gastric cancer.

**Methods:**

Studies assessing the prognostic role of the pretreatment CAR in patients with gastric cancer were searched from PubMed, Embase, and Cochrane Library up to June 6, 2019. Pooled hazard ratios (HRs) for overall survival (OS), recurrence-free survival (RFS), and cancer-specific survival (CSS) were estimated using a fixed-effects model.

**Results:**

Eight observational studies including 3102 patients were enrolled in this meta-analysis. The pooled result showed that patients with a high CAR had worse OS (pooled HR = 1.87; 95% confidence interval (CI) = 1.55–2.26; *P* < .001). Results from subgroup analyses indicated that patient country, adjuvant chemotherapy rate, and CAR cut-off value could not affected the property of the correlation (*P* < .001). However, the intensity of the correlation was affected by these factors. In addition, patients with a high CAR had significantly worse RFS (pooled HR = 2.11; 95% CI = 1.41–3.15; *P* < .001) and CSS (HR = 1.59; 95% CI = 1.08–2.35; *P* = .019).

**Conclusion:**

A high pretreatment CAR was significantly associated with poor survival for patients with gastric cancer. The prognostic significance of the pretreatment CAR in gastric cancer is need to be confirmed by clinical trials of large sample size.

## 1 Introduction

Gastric cancer is a major health issue worldwide due to high morbidity and mortality.^[1]^ In China, gastric cancer is the third highest cause of cancer death in men and the second in women.^[2]^ With the rise of targeted therapy and immunotherapy in recent years, the prognosis of gastric cancer has been greatly improved. For example, the current 5-year survival rate for patients with early gastric cancer is 80% to 95%. However, patients with advanced gastric cancer have a five-year survival rate of only 2%.^[3]^ At present, the prognosis of patients with gastric cancer is mainly predicted according to traditional tumor-node-metastasis (TNM) stage. This prediction method has limited accuracy and cannot accurately stratify the patient's prognosis. Because of this, people have been working on exploring new biomarkers with high sensitivity and specificity that can accurately access the long-term prognosis of patients with gastric cancer.

C-reactive protein (CRP) is an important acute-phase response protein synthesized by liver cells and is one of the most sensitive indicators of inflammation.^[4]^ Tumor tissue can trigger the body's inflammatory response, so CRP of tumor patients is often elevated.^[4]^ Albumin (Alb) is synthesized by the liver and is the main component of human serum total protein.^[5]^ Albumin plays an important role in maintaining blood colloid osmotic pressure, transporting metabolites, and reflecting nutritional status.^[5]^ Because tumor patients have poor nutritional status, their serum albumin levels are often low. As an indicator that can reflect both inflammatory and nutritional status, CRP/Alb ratio (CAR) is elevated in most tumor patients.^[6]^ A higher CAR indicates a worse general condition for tumor patients.^[7]^

The association between CAR and prognosis has been validated in a variety of tumors.^[8–10]^ Kudou et al^[11]^ and Liu et al^[12]^ found that patients with high pretreatment CAR levels had worse overall survival (OS), recurrence-free survival (RFS), and cancer-specific survival (CSS) in gastric cancer. However, the sample size contained in these studies was relatively small, and there were some differences in the results. To obtain an accurate result based on a larger sample size, we conducted this meta-analysis.

## 2 Methods

### 2.1 Search strategy

This meta-analysis was conducted in compliance with the Systematic Reviews and Meta-Analyses (PRISMA) guidelines.^[13]^ Literatures were searched from PubMed, Embase, and the Cochrane Library (last update by June 6, 2019) using the medical subject heading (MeSH) terms “C-reactive protein”, “albumins”, and “stomach neoplasms”. There were no language restrictions during the search.

### 2.2 Inclusion criteria

The inclusion criteria were as follows:

(1) retrospective studies investigated the role of CAR in prognostic evaluation of gastric cancer;
(2) the CAR was calculated with serum CRP and albumin levels before chemotherapy and surgery;
(3) the hazard ratio (HR) and 95% confidence interval (CI) of CAR could be extracted.

### 2.3 Data extraction

Some important study characteristics were extracted, including the first author's surname, publication year, country, sample size, patients’ ages, tumor location, proportion of patients receiving adjuvant chemotherapy, analysis method, CAR cut-off value, length of follow-up, TNM stage, and HRs and the corresponding 95% CIs of CAR. Because multivariate analysis considers the confounding factors, it is preferred to be adopted over univariate analysis.

### 2.4 Quality assessment

The Newcastle-Ottawa quality assessment scale (NOS)^[14]^ was used to assess the quality of each study. Quality assessment scores range from 0 (lowest) to 9 (highest), and a score of 6 or higher indicates high quality.

### 2.5 Statistical analysis

The optimal cut-off value obtained from the receiver operating characteristic (ROC) curve was used to distinguish the CAR level in all included studies. When HR and 95% CI were not reported, we estimated them based on data extracted from Kaplan–Meier survival curves.^[15]^ We assessed heterogeneity using the chi-square test (assessing the *P* value) and *I*^2^ statistic.^[16,17]^ There was no heterogeneity only when the *P* value > .05 and the *I*^2^ < 50%. If there was no heterogeneity, a fixed-effects model (the Mantel–Haenszel method) was used,^[18]^ otherwise a random effect model (DerSimonian–Laird method) was used.^[19]^ Begg and Egger tests (assessing the *P* value) and a funnel plot were used to estimate the publication bias. An asymmetric funnel plot and/or a *P*-value < .05 indicated publication bias. At this point, we adjusted the publication bias using the “Trim and Fill” method.^[20]^ STATA version 12.0 (Stata Corporation, College Station, TX) was used to perform analyses or generate figures. A *P*-value < .05 indicated statistical significance.

All analyses were based on previous published studies; thus, no ethical approval or patient consent was required.

## 3 Results

### 3.1 Study characteristics

**Figure 1:**
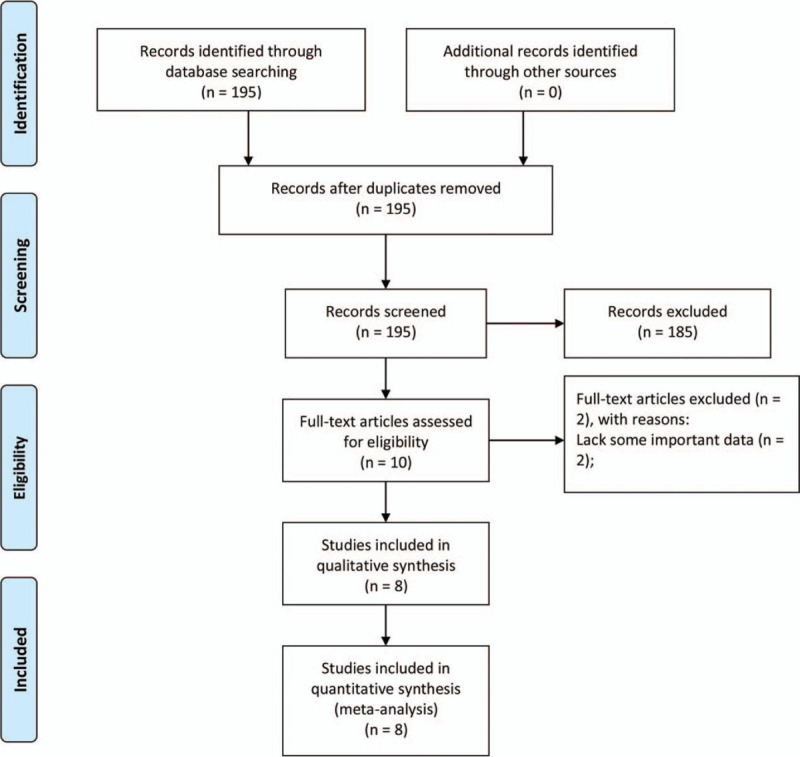
Flow diagram of the study selection process.

By searching the MeSH terms, 195 studies were retrieved from the databases. After preliminary screening, 185 studies were excluded. The full text of the remaining 10 studies was reviewed, and two were excluded due to lack of important data. Finally, a total of 3102 patients from eight studies were included in this meta-analysis (Fig. 1).^[11,12,21–26]^ The mean NOS score for the eight studies was 6.75, ranging from 5 to 9.

Table 1 shows the main characteristics of the included studies. The number of patients in each study varied between 114 and 688. All patients were from China or Japan. Seven studies reported the proportion of patients receiving adjuvant chemotherapy, which ranged from 14.1% to 100%. In most studies, the location of the gastric cancer included the upper, middle, and lower thirds of the stomach. The optimal CAR cut-off values determined according to their respective ROC curves were used in all studies. The HRs for OS were reported directly in five studies and were estimated indirectly in one study. The HRs for RFS in three studies were all reported directly. Only one study focused on patients’ CSS. In addition, none of the patients in the included studies received preoperative or adjuvant radiotherapy.

**Table 1:**
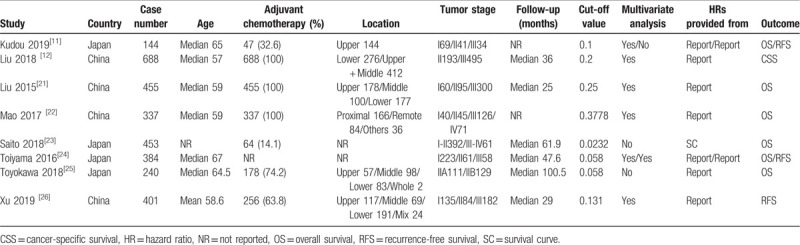
Main characteristics of all studies included in the meta-analysis.

### 3.2 Overall survival

**Figure 2:**
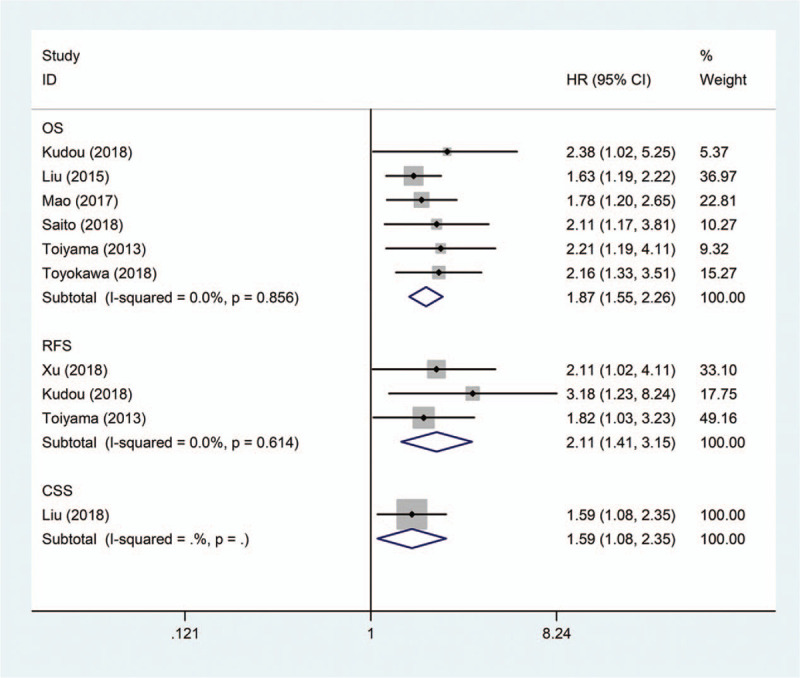
Forest plots of studies evaluating the hazard ratio for the survival of gastric cancer patients with a high C-reactive protein/albumin.

The main results of the pooled analysis were showed in Table 2. Six studies including 2013 patients provided HRs and 95% CIs regarding the relationship between the CAR and OS in patients with gastric cancer. A fixed-effects model was used to pool the HRs of these studies because there was no significant heterogeneity (*I*^2^ = 0%, *P* = .856). The pooled result showed that patients with a high CAR had worse OS (pooled HR = 1.87; 95% CI = 1.55–2.26; *P* < .001) (Fig. 2).

**Table 2:**
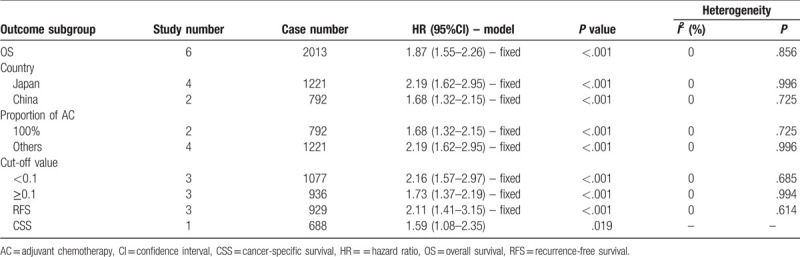
Pooled hazard ratios for patients’ survival according to subgroup analyses.

**Figure 3:**
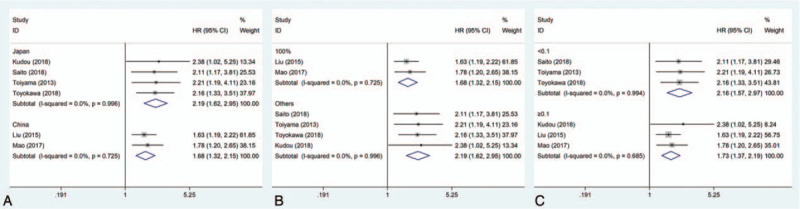
Forest plots of studies evaluating the hazard ratio for the overall survival of gastric cancer patients with a high C-reactive protein/albumin ratio divided by country (A), adjuvant chemotherapy rate (B), or cut-off value (C).

Subgroup analyses were subsequently performed to investigate the effects of different clinical characteristics on the pooled HR. The results showed that patient country, proportion of adjuvant chemotherapy, and CAR cut-off value could not affected the property of the association between the CAR and OS (*P* < .001, Table 2). However, the association between a high CAR and poor OS was stronger in Japanese patients than in Chinese patients (Table 2; Fig. 3A). In addition, the association between a high CAR and poor OS in the subgroup with a 100% adjuvant chemotherapy rate was comparatively lower (Table 2; Fig. 3B). When the CAR cut-off value was less than 0.1, the association between a high CAR and poor OS in patients with gastric cancer appeared comparatively higher (Table 2; Fig. 3C).

**Figure 4:**
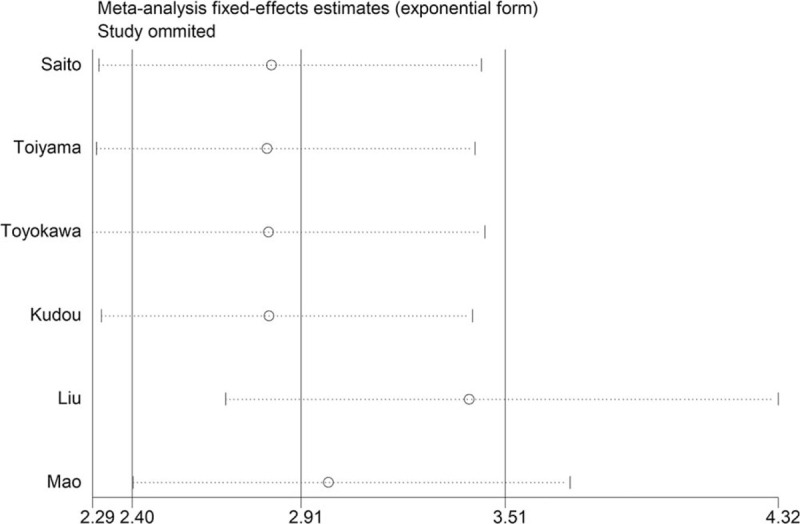
Sensitivity analysis of studies evaluating the relationship between the C-reactive protein/albumin ratio and overall survival in patients with gastric cancer.

Because there was no significant heterogeneity, a fixed-effects model was also used for the sensitivity analysis. The results showed that the result pattern was not obviously affected by any single study (Fig. 4). In addition, the results from the meta-regression showed that patient country, proportion of adjuvant chemotherapy, and CAR cut-off value did not affected the pooled effect size (*P* = .254, *P* = .254, and *P* = .338, respectively).

**Figure 5:**
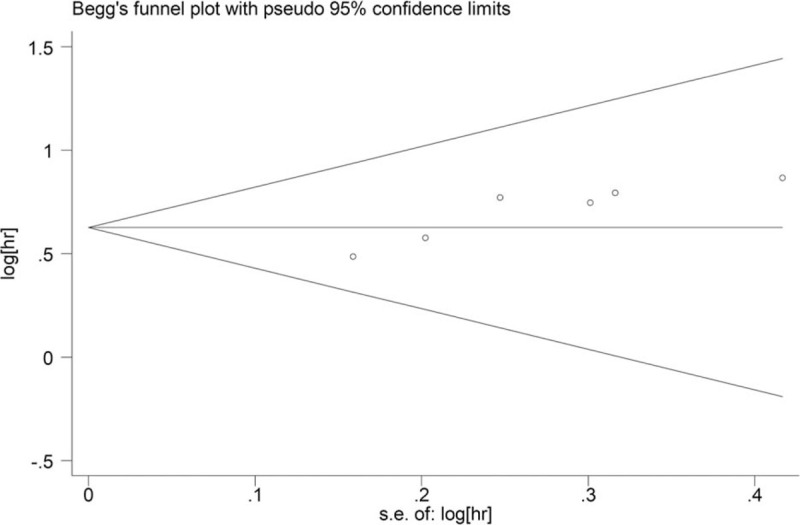
Funnel plot to examine publication bias among studies evaluating the relationship between the C-reactive protein/albumin ratio and overall survival in patients with gastric cancer.

Because the funnel plot was asymmetrical (Fig. 5), and the *P* value for the Egger test was .007, there was a publication bias in this meta-analysis. Therefore, a “Trim and Fill” method under a fixed-effects model was used. The adjusted pooled HR for OS was 1.74 (95% CI = 1.47–2.05; *P* < .001).

### 3.3 Recurrence-free survival

Three studies including 929 patients provided the HR and 95% CI regarding the correlation between the CAR and RFS in patients with gastric cancer. We used a fixed-effects model to pool the HRs because there was no significant heterogeneity in these studies (*I*^2^ = 0%, *P* = .614). The pooled result showed that patients with a high CAR had worse RFS (pooled HR = 2.11; 95% CI = 1.41–3.15; *P* < .001) (Table 2; Fig. 2).

### 3.4 Cancer-specific survival

Only one study, which including 688 patients and was carried out by Liu et al,^[12]^ provided data regarding the association between the CAR and patients’ CSS in gastric cancer. They demonstrated through multivariate analysis that patients with a high CAR had worse CSS (HR = 1.59; 95% CI = 1.08–2.35; *P* = .019) (Table 2; Fig. 2).

## 4 Discussion

In recent years, several biological indicators reflecting the systemic inflammatory response have shown an important role in the prognostic evaluation of many types of tumor.^[27–30]^ The CAR is the ratio of serum CRP level to serum albumin level. The serum CRP level is related to the activity of tumors because specific antigens on the surface of tumor cells can induce the anti-tumor immune response, and tumor cells also produce inflammatory proteins to promote the secretion of CRP.^[31–33]^ Low-serum albumin level indicating that the body is in a state of malnutrition, which has been shown to be associated with poor outcomes in gastric cancer.^[34,35]^ Gastric cancer often causes difficulty in eating and/or digestive dysfunction, so the proportions of malnutrition and cachexia in patients with gastric cancer are comparatively high. In addition, patients with gastric cancer usually receive multiple treatments, including surgery, radiation therapy, and chemotherapy. These treatments can cause inappetence and abnormal protein metabolism, leading to intensified malnutrition. Therefore, the CAR that can comprehensively reflect inflammation and nutritional status is very suitable for predicting the prognosis of gastric cancer.

Although no study has clearly shown that CAR has an impact on tumorigenesis and metastasis, it may indirectly affect tumorigenesis and metastasis through serum CRP and albumin. A high CAR represents elevated serum CRP concentration and/or low-serum albumin level. Elevated serum CRP concentrations are often accompanied by increased serum concentrations of vascular endothelial growth factor, contributing to tumor formation and progression.^[36]^ Albumin is the most commonly used indicator in the clinical evaluation of patients’ nutritional status.^[37]^ Low-serum albumin levels suggest malnutrition, which can alter the tumor cell biology in the tumor microenvironment and damage the immune system to promote tumor growth and metastasis.^[38,39]^ Therefore, a high CAR indicates that the body is in a favorable state for tumorigenesis and metastasis, which should be given more attention in clinical practice. The Glasgow Prognostic Score (GPS) and modified GPS (mGPS) are two other biological indicators calculated according to serum CRP and albumin concentrations, which can also be used for predicting the prognosis of gastric cancer.^[40,41]^ However, compared to CAR, GPS, and mGPS applications are more complicated because they are based on scores converted from serum CRP and albumin concentrations. In addition, a previous study used a stepwise regression method to find main factors affecting prognosis, and the results showed that CAR is more suitable for building the best-fit prediction model than GPS or mGPS.^[42]^ Another previous study compared the area under the curve (AUC) of CAR, GPS, and mGPS.^[21]^ The results show that the AUC of CAR is the largest, suggesting that CAR is more suitable for prognostic evaluation.^[21]^

This meta-analysis, including the data of 3102 patients enrolled in eight studies, strongly suggested that patients with a high pretreatment CAR have poor outcomes in gastric cancer. Results from subgroup analyses indicated that patient country, adjuvant chemotherapy rate, and CAR cut-off value affected the intensity of the association between pretreatment CAR and OS but did not affect the property of the association. For example, the association between a high CAR and poor OS was comparatively lower in the Chinese or the 100% adjuvant chemotherapy rate subgroups. Remarkably, the two studies in the Chinese subgroup were the same as the two studies in the 100% adjuvant chemotherapy rate subgroup. Therefore, it is still not clear whether the difference between the subgroups is caused by nationality or by chemotherapy. A randomized controlled trial may be needed to find the answer.

This meta-analysis is the first study to systematically review and analyze the prognostic role of the CAR in gastric cancer. There are still some deficiencies in this meta-analysis. First, this study included only eight eligible studies, which resulted in relatively insufficiency data in the subgroup analyses. For example, we failed to perform a subgroup analysis of pathological types. Second, all included patients were from China or Japan, so the findings of this may be more suitable for Asian patients. For Caucasian gastric cancer patients, the prognostic role of the CAR remains unknown, but the prognostic role of serum CRP and albumin has been clarified.^[43,44]^ Ilhan et al^[43]^ reported that the level of serum CRP in Caucasian gastric cancer patients was significantly higher than that in healthy persons. Palaj et al^[44]^ found that high-serum albumin levels were associated with more lymph node involvement and worse OS in Caucasian gastric cancer patients. Similar findings have been observed in Asian patients,^[45,46]^ suggesting that serum CRP and albumin play a similar role in Asian patients as they do in Caucasian patients. Therefore, we speculate that a high CAR is also associated with poor prognosis in Caucasian gastric cancer patients. Third, some included studies provided only the HRs from univariate analysis, so the effect size may be overestimated. Finally, several HRs were estimated according to the data extracted from the survival curves, whereas there is error between estimated HRs and actual HRs.

In conclusion, the pretreatment CAR is convenient and precise for predicting the prognosis of patients with gastric cancer. The pretreatment CAR may also be of guiding significance to nutritional support and anti-inflammatory treatment for patients with gastric cancer. The findings of this study are still need to be confirmed by clinical trials of large sample size.

## Author contributions

**Conceptualization:** Changping Wu.

**Data curation:** Xuanxuan Yang, Luo Zhang.

**Formal analysis:** Xuanxuan Yang, Xing Song, Luo Zhang.

**Methodology:** Xing Song, Luo Zhang.

**Resources:** Changping Wu.

**Software:** Xuanxuan Yang, Xing Song, Luo Zhang.

**Supervision:** Changping Wu.

**Writing – original draft:** Xuanxuan Yang.

**Writing – review & editing:** Changping Wu.
